# The short-term and long-term influence of a single pain education session on knowledge, attitudes and beliefs of doctor of physical therapy students

**DOI:** 10.1186/s12909-025-07816-1

**Published:** 2025-09-25

**Authors:** Sarah Case-Morris, William H. Suits, Tzu-Chieh Liao, Lindsey A. Fox

**Affiliations:** 1 Physical Therapy Department, University of Michigan-Flint, Flint, MI USA; 2 University of Louisville Health, Louisville, KY USA; 3 Department of Radiology, University of Michigan, Ann Arbor, MI USA

**Keywords:** Physical therapists, Pain, Education, Knowledge, Beliefs

## Abstract

**Background:**

To determine the effectiveness of a brief, structured educational session, based on the International Association for the Study of Pain (IASP) core competencies, in improving knowledge, attitudes and beliefs of physical therapy students towards pain and to determine whether those changes are sustained one year later.

**Methods:**

A longitudinal prospective cohort study was conducted within an entry-level Doctor of Physical Therapy (DPT) program. Participants (*n* = 172) in the first or second year of the DPT program completed the Revised Neurophysiology of Pain Questionnaire (rNPQ) and the Pain Attitudes and Beliefs Scale for Physiotherapists (PABS-PT).

**Results:**

Eighty-two participants (age, 25.00 ± 3.25 years; 59 females) completed the pre survey resulting in a 47.6% response rate. Both the post survey and the one year follow up were completed by 57 students. Participants exhibited lower PABS-Bio scores directly following the education session as compared to baseline (*p* < 0.001), but not at one year follow-up (*p* = 0.163). Participants exhibited higher PABS-Psy scores following the session (*p* < 0.001) and at one year follow-up (*p* = 0.001). Participants exhibited improved rNPQ scores following the session (*p* = 0.043) and at one year follow-up (*p* < 0.001).

**Conclusions:**

Significant changes occurred in knowledge, attitudes, and beliefs regarding pain immediately after an educational session. Pain knowledge increased one year later, but sustained changes in attitudes and beliefs regarding pain were mixed at one year follow-up. A single education session alone may be insufficient to change attitudes and beliefs in the long term. It is not known if a more frequent mode of delivery, or content threaded through several courses across the curriculum may lead to greater changes long-term. Further research determining the mode, frequency, and integration of pain-related content with other content in a DPT curriculum is needed.

## Background

Chronic pain affects up to 37% of the entire population, and management of chronic pain results in over $100 billion US dollars of medical spending annually [1–3]. Treatment provided to people with chronic pain yields various degrees of efficacy, and it is generally recommended that healthcare providers take a biopsychosocial approach to address the complex nature of pain [1, 4, 5]. Due to the massive burden chronic pain has on society, there are calls to improve education for those who provide healthcare to individuals with chronic pain [3, 4]. 

Clinical practice guidelines for the management of chronic pain recommend non-pharmacological interventions as a first-line approach, and physical therapists (PTs) are often heavily involved in the nonpharmacologic management of chronic pain [6–9]. Training for the management of chronic pain is inconsistent, ranging from 5 to 115 hours in an entry-level physical therapy curriculum [5]. Less than 50% of physical therapy students demonstrate awareness of best practice clinical practice guidelines for the management of chronic pain [5]. This may result in physical therapists managing chronic pain from a traditional biomedical approach, which is in contrast to the currently recommended biopsychosocial approach [10]. 

The International Association for the Study of Pain (IASP) published a recommendation for pain education which endorses a biopsychosocial approach for physical therapist education [4, 5]. This recommendation lists out specific domains of learning and core competencies for students. There is conflicting evidence regarding the timing or mode of education for these core competencies and contemporary clinical practice guidelines on pain management [11–16]. In particular, it is unclear if stand-alone courses or modules on pain are as effective as integrated content for facilitating sustained improvements of knowledge, attitudes, and beliefs about pain in entry-level physical therapy programs [15, 16]. 

Therefore, the present study seeks to determine the effectiveness of a brief, structured educational session, based on IASP core competencies, in improving knowledge, attitudes, and beliefs of physical therapy students towards pain and to determine whether those changes are sustained one year later.

## Methods

### Design

A longitudinal prospective cohort study was conducted at a single university within an entry-level DPT program. In order to meet multiple student learning objectives, DPT students were required to attend the six-hour pain educational session based on the IASP recommendations. Prior to the educational session, students were invited to participate in the research survey regarding their knowledge, attitudes, and beliefs towards pain and take the pre-survey. Participation was voluntary and students completed an informed consent prior to taking the survey acknowledging that their grades would not be affected by the research study.

The survey was administered using Qualtrics Online Survey Software (Qualtrics LLC, Provo, Utah, USA) and consisted of demographic questions (age and gender), the revised Neurophysiology of Pain Questionnaire (rNPQ) and the Pain Attitudes and Beliefs Scale for Physiotherapists (PABS-PT). The survey also included items related to participants’ past experiences, such as personal or familial chronic pain history, experience in treating or observing patients with chronic pain, outside entry-level training in pain, and memberships in pain organizations. Although these experience-related items were not included in the statistical analysis, they were collected to provide descriptive information about the participants’ characteristics. Participants were asked to create a unique identifier using the first two letters of their mother’s name and last two digits of their cell number. No other identifying information was collected during the study. Nevertheless, some participant data could not be matched over time due to errors in participants recalling their unique identifiers.

### Measures

Knowledge of pain was assessed using the rNPQ and attitudes and beliefs were assessed using the PABS-PT [17, 18]. These instruments were selected based on prior research demonstrating a positive relationship between the knowledge, attitudes, and beliefs of a health care provider and how that health care provider addressed pain [10, 13, 19, 20]. The rNPQ and PABS-PT have also been previously utilized in studies of physical therapy students’ pain knowledge, attitudes and beliefs [12, 15, 16, 25, 27]. 

The rNPQ consists of 12 close-ended items that assess an individual’s understanding of pain neurobiology and knowledge of pain. The rNPQ is scored by the number of correct items where higher scores indicate increased knowledge. The rNPQ has demonstrated acceptable reliability and construct validity [17, 21]. 

The PABS-PT was developed to evaluate the attitudes and beliefs of physical therapists in how they treat low back pain [18, 22]. The PABS-PT consists of 19 questions with two subscales: biomedical and biopsychosocial. The questions distinguish between biomedical and biopsychosocial orientation using a 6-point Likert scale ranging from strongly disagree to strongly agree [18, 22]. The biomedical subscale (PABS-Bio) consists of 10 questions with a maximum possible score of 60 while the biopsychosocial subscale (PABS-Psy) consists of 9 questions with a maximum possible score of 54. Higher scores on each subscale indicate stronger treatment orientation in that direction [18, 22]. The PABS-PT has acceptable construct validity and reliability [23]. 

### Educational session

Faculty teaching in the entry-level physical therapy program developed an introductory educational session to address selected IASP core competencies for pain education intended to be delivered in the first academic year of the DPT curriculum. However, due to COVID-19 restrictions, the lecture-based educational session was delivered virtually in 2021 to both first-year and second-year cohorts, and again in 2022 to only first-year students. Each student who participated in the session did so only once. Consequently, the study participants included a mix of students in either their first or second year of the entry-level DPT program. Content regarding the epidemiology of pain, pain mechanisms, pain neurophysiology, pain assessment, pain management and evidence-based interventions in acute and outpatient settings was included. The session also included a brief introduction to Pain Neuroscience Education (PNE).

### Analysis

The descriptive analysis summarized categorical variables as N (%) and continuous variables as mean ± standard deviation. Linear mixed models (LMMs) were used to determine the differences in PABS-Bio, PABS-Psy, and rNPQ prior to, directly following, and one year after the pain education session. Time was modeled as a fixed effect to examine its impact on the dependent variable while subject ID (available for a subset of participants) was included as a random effect to capture within-subject variability for matched responses. Using LMMs allowed unmatched data points to be treated as independent observations while capturing the average trend through the fixed effect for time [24]. In addition, the response rate varied across the three time points, and participants who completed the initial survey did not necessarily complete subsequent surveys, or vice versa. LMMs robustness to unbalanced datasets ensured accurate analysis despite differing numbers of observations per time point. If a significant main effect was found (time), post hoc analysis was performed with Bonferroni correction to examine where the changes occurred. An alpha level of 0.05 was used for statistical significance. All statistical analyses were conducted using SPSS version 28.0.0.0.

## Results

Out of the 172 students who attended the education session over two years, 82 (age, 25.00 ± 3.25 years; 59 females) physical therapy students completed the pre survey (baseline) resulting in a 47.6% response rate. The majority of participants were first-year students who reported having a personal or familial history of chronic pain (80.5%) and prior experience in treating or observing patients with chronic pain–either frequent or infrequent (95.2%). However, most participants did not have outside training in chronic pain management (89.0%) and were not members of any pain-specific groups or organizations (91.5%). Subject characteristics and experiences at baseline are presented in Table 1.

**Table 1:** Characteristics of physical therapy students that participated in the survey prior to an education seminar

	(*n* = 82)
Age, years (mean ± SD)	25.00 ± 3.25
Gender, n(%)	
Females	59 (71.9%)
Males	23 (28.1%)
Year in Program, n (%)	
First-Year	66 (80.5%)
Second-Year	16 (19.5%)
Personal or Familial Chronic Pain History, n (%)	
Yes	65 (79.3%)
No	17 (20.7%)
Experience in Treating or Observing Patients with Chronic Pain, n (%)	
Frequent	39 (47.6%)
Infrequent	39 (47.6%)
Never	4 (4.8%)
Outside Entry-Level Training, n (%)	
Yes	9 (11.0%)
No	73 (89.0%)
Membership Status, n (%)	
Yes	7 (8.5%)
No	75 (91.5%)

Following the education session, 57 students completed the post-survey. At the one-year follow-up, another 57 students completed the survey. Only a small subset of participants (*n* = 20) could be confidently matched across all three time points due to errors in recalling unique identifiers. LMMs show that the main effect (time) existed in all three outcome measures (all *p* < 0.001). Students’ pain knowledge, attitudes and beliefs prior to, directly after, and one year following the pain education seminar are shown in Table 2.

**Table 2:** Students’ pain knowledge, attitudes and beliefs prior to, directly after, and one year following a pain education seminar

Group Means (95% CI)			
	Pre-Session (n=82)	Post-Session (n=57)	1Y FU (n=57)
PABS			
Biomedical Scale	41.83 (40.07, 43.59)	32.75 (30.74, 34.76)	39.40 (37.45, 41.52)
Biopsychosocial Scale	40.40 (39.47, 41.33)	44.30 (43.22, 45.37)	42.79 (41.69, 43.89)
rNPQ	7.45 (7.02, 7.87)	8.03 (7.55, 8.50)	9.91 (9.41, 10.40)

Within Group Means (95% CI) (Between-Time)			
	Pre – Post	Pre – 1Y FU	Post – 1Y FU
PABS			
Biomedical Scale	-9.08* (-6.65, -11.51)	-2.34 (5.27, 0.58)	6.73* (3.62, 9.84)
Biopsychosocial Scale	3.90* (2.54, 5.25)	2.39* (0.79, 3.99)	-1.50 (-3.22, 0.20)
rNPQ	0.57* (0.01, 1.14)	2.46* (1.75, 3.16)	1.88* (1.14, 2.62)

Data presented as mean (95% confidence interval)

*1Y FU* One year follow-up, *PABS* Pain Attitudes and Beliefs Scale, *rNPQ* Revised Neurophysiology of Pain Questionnaire

* indicates significant differences (*p*<0.05)

**Fig. 1:**
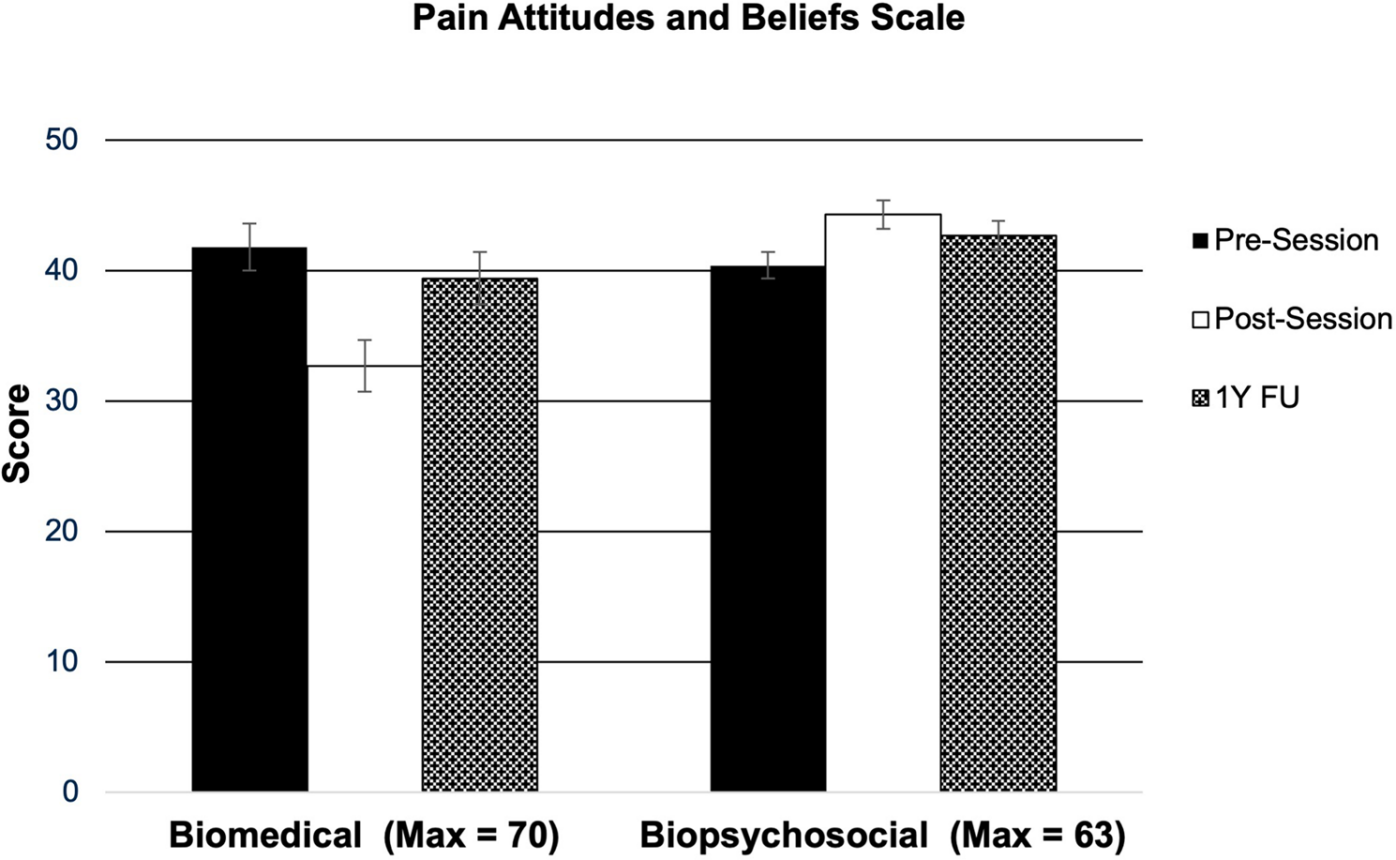
Pain attitudes and beliefs scale

**Fig. 2:**
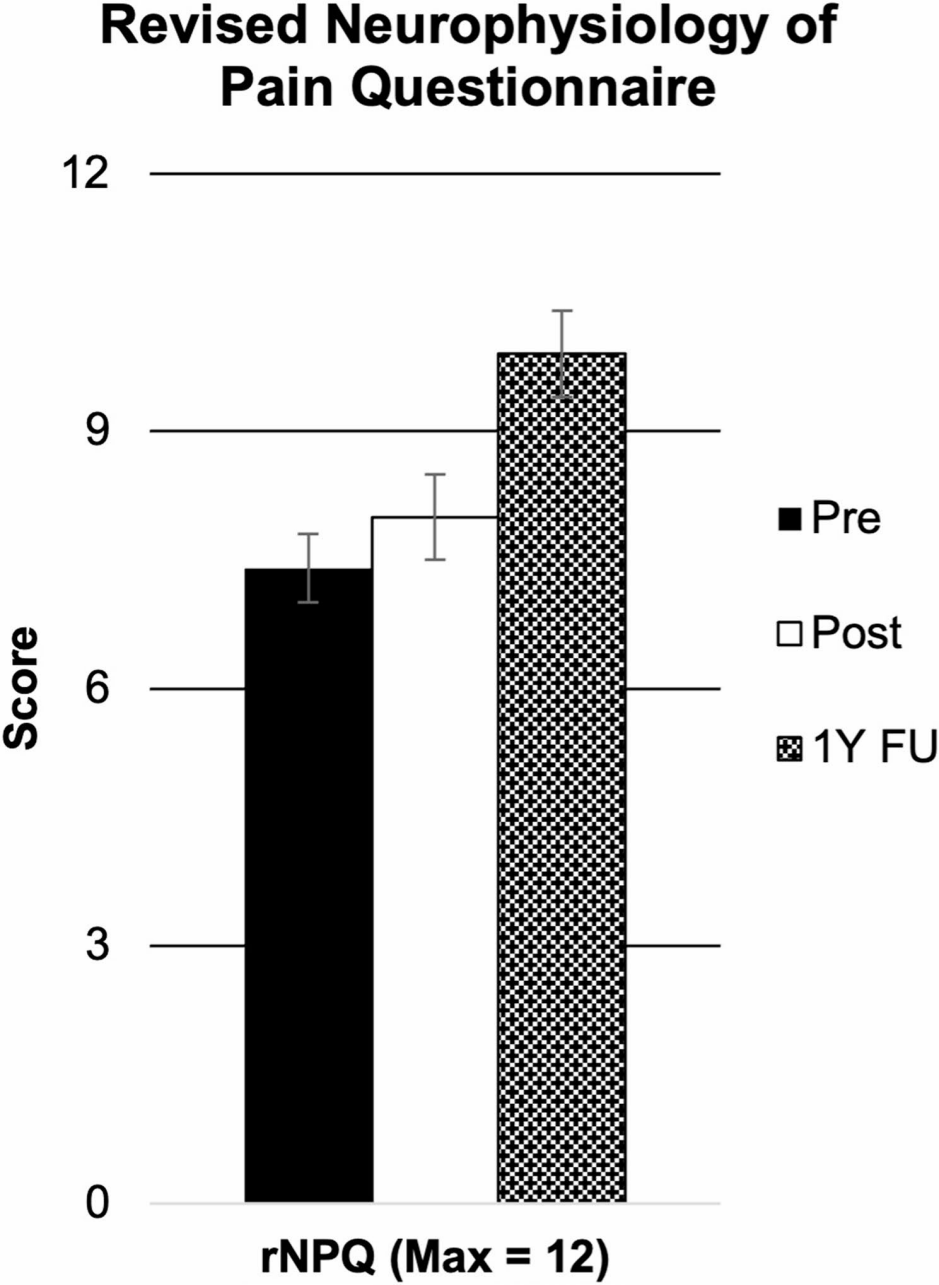
Revised neurophysiology of pain questionnaire

As shown in Fig. 1, post-hoc analysis revealed that students exhibited lower PABS-Bio scores directly following the education session as compared to baseline (*p* < 0.001), but not at one year follow-up (*p* = 0.163). Students exhibited higher PABS-Psy scores following the session (*p* < 0.001) and at one year follow-up (*p* = 0.001). Lastly, students exhibited improved rNPQ scores following the session (*p* = 0.043) and at one year follow-up (*p* < 0.001) as shown in Fig. 2.

## Discussion

The purpose of this study was to describe the effectiveness of a brief educational session on improving students’ knowledge, attitudes and beliefs about pain and to compare the short-term and long-term changes in knowledge, attitudes, and beliefs regarding pain in physical therapy students. Previous research shows that knowledge, attitudes, and beliefs appear to change short-term after education interventions in physical therapy students in the United States [11–13, 15, 16, 25]. However, prior to this study, less was known about whether these changes could be sustained. The results of this study demonstrate significant changes in knowledge, attitudes, and beliefs regarding pain immediately after an educational session. Pain knowledge increased one year later, but sustained changes in attitudes and beliefs regarding pain were mixed at one year follow-up. Therefore, any changes in knowledge, attitudes and beliefs in the long term cannot be attributed to the single-session education intervention. Further research is needed to investigate factors associated with long-term changes in attitudes and beliefs regarding pain in physical therapy students. Confounding factors such as clinical education experiences, attitudes and beliefs of clinical instructors, personal experiences and subsequent curricular content may limit the generalizability of these findings.

Calls for revamping physical therapy curricula to include contemporary pain content focuses heavily on the recommended content and sparsely on timing or mechanisms of delivery [4, 26]. The explicit goal of the proposed curricular changes in physical therapy education is to enhance pain management strategies and improve patient outcomes in the real world setting. Implicitly, the knowledge, attitudes, and beliefs of the future physical therapists may impact the quality of care they deliver. The results of this study show that while an educational session based on the recommended IASP core competencies facilitates short-term favorable changes in the knowledge, attitudes, and beliefs regarding pain in physical therapy students, these changes may lessen over a longer term. Therefore, if the goal is to encourage changes in knowledge, attitudes, and beliefs in developing clinicians, a single education session alone may be insufficient, even if it is focused on the recommended content. It is not known if a more frequent mode of delivery, and/or content threaded through several classes across the curriculum may lead to greater outcomes long-term. Further research determining the mode, frequency, and integration of pain-related content with other content in a PT curriculum is needed.

The duration of the educational session in this study was a mere six hours, considerably shorter than most previously studied pain science educational experiences. However, the acute changes in knowledge, attitudes, and beliefs found in this study were comparable to longer duration educational experiences [12, 25, 27]. On the surface, this suggests that the content is more important than the duration of the educational session, but the impact of a single-session on long-term knowledge retention and changes in attitudes and beliefs is not substantiated by this study. It is possible that longer educational sessions, and/or strategies to embed content over the course of an entire curriculum may foster greater sustainment of knowledge, attitudes and beliefs in the long-term [26, 27]. Despite its limitations, the brief, single-session offers a practical and cost-effective approach that may be particularly useful for institutions with limited resources or constraints that prevent substantial curriculum changes. While attitudes and beliefs about pain may influence treatment behaviors, it is not known what type of educational intervention may influence clinical behaviors in a manner which improves outcomes for patients with chronic pain [20, 28, 29]. Research comparing patient-reported outcomes to clinician behaviors, attitudes and beliefs may be more impactful.

This study was performed at a single educational institution in the United States. While there are at least some commonalities across all accredited physical therapy education programs in the United States in terms of educational content, there are numerous differences in timing and delivery of curricula which potentially affect knowledge outcomes. This limits the generalizability of the results of this study. In addition, there was no control group in this study, and there was no control for content covered in other aspects of the curriculum or in the clinical education experiences. It is unclear how other portions of the curriculum may have influenced the results found in this study. An additional limitation was the inability to reliably match subjects over time. Only a small number of matched data sets were available, which was insufficient for longitudinal within-subject analysis. Therefore, data from all time points were pooled and analyzed using LMMs, a reasonable approach given the design, but this limits the interpretation of individual change over time. As such, cautions should be made when making comparisons regarding the long-term changes.

Future research could include larger cohorts or cross-sectional studies of students from multiple programs or investigations looking into optimal mode, frequency, and timing of content in entry-level DPT curricula. Whether knowledge, attitudes, and beliefs change based on external influences, such as additional training and clinical exposures may be worth exploring as well [30]. Finally, future studies could explore the effect of educational interventions on clinical behaviors and patient outcomes.

## Conclusions

The results of this study demonstrate that a single educational session on pain significantly influences knowledge, attitudes, and beliefs of physical therapy students only in the short-term, yet other factors may influence long-term changes. An approach that goes beyond a single educational session may be warranted if physical therapy educators wish to facilitate sustained changes in knowledge, attitudes, and beliefs regarding pain.

## Data Availability

No datasets were generated or analysed during the current study.

## Abbreviations

DPT: Doctor of physical therapy
IASP: International Association for the Study of Pain
LMMs: Linear mixed models
PABS-Bio: PABS-PT, biomedical subscale
PABS-PT: Pain attitudes and beliefs scale for physiotherapists
PABS-Psy: PABS-Psy, biopsychosocial subscale
PTs: Physical therapists
rNPQ: Revised neurophysiology of pain questionnaire
